# Cerebromicrovascular senescence in vascular cognitive impairment: does accelerated microvascular aging accompany atherosclerosis?

**DOI:** 10.1007/s11357-025-01621-w

**Published:** 2025-03-21

**Authors:** Anna Ungvari, Ádám Nyúl-Tóth, Roland Patai, Boglarka Csik, Rafal Gulej, Dorina Nagy, Santny Shanmugarama, Zoltán Benyó, Tamas Kiss, Zoltan Ungvari, Anna Csiszar

**Affiliations:** 1 Institute of Preventive Medicine and Public Health, Semmelweis University, Budapest, Hungary; 2 Vascular Cognitive Impairment, Neurodegeneration and Healthy Brain Aging Program, Department of Neurosurgery, University of Oklahoma Health Sciences Center, Oklahoma City, OK USA; 3 Oklahoma Center for Geroscience and Healthy Brain Aging, University of Oklahoma Health Sciences Center, Oklahoma City, OK USA; 4 International Training Program in Geroscience, Doctoral College/Institute of Preventive Medicine and Public Health, Semmelweis University, Budapest, Hungary; 5 Institute of Translational Medicine, Semmelweis University, Budapest, Hungary; 6 Cerebrovascular and Neurocognitive Diseases Research Group, HUN-REN, Semmelweis University, Budapest, Hungary; 7 Pediatric Center, Semmelweis University, Budapest, Hungary; 8 Stephenson Cancer Center, University of Oklahoma, Oklahoma City, OK USA; 9 Department of Health Promotion Sciences, College of Public Health, University of Oklahoma Health Sciences Center, Oklahoma City, OK USA; 10 International Training Program in Geroscience, Doctoral College/Institute of Translational Medicine, Semmelweis University, Budapest, Hungary

**Keywords:** Aging, Atherosclerosis, Atherogenesis, Cerebral circulation, Arteriosclerosis, Peripheral artery disease, Oxidative stress, Inflammation, Senolytics, Endothelial biomarkers

## Abstract

Vascular cognitive impairment (VCI) is a leading cause of age-related cognitive decline, driven by cerebrovascular dysfunction and cerebral small vessel disease (CSVD). Emerging evidence suggests that cerebromicrovascular endothelial senescence plays an important role in the pathogenesis of VCI by promoting cerebral blood flow dysregulation, neurovascular uncoupling, blood–brain barrier (BBB) disruption, and the development of cerebral microhemorrhages (CMHs). This review explores the concept of cerebromicrovascular senescence as a continuum of vascular aging, linking macrovascular atherosclerosis with microvascular dysfunction. It examines the mechanisms by which endothelial senescence drives neurovascular pathology and highlights the impact of cardiovascular risk factors in accelerating these processes. We examine preclinical and clinical studies that provide compelling evidence that atherosclerosis-induced microvascular senescence exacerbates cognitive impairment. In particular, findings suggest that targeting senescent endothelial cells through senolytic therapy can restore cerebrovascular function and improve cognitive outcomes in experimental models of atherosclerosis. Given the growing recognition of microvascular senescence as a therapeutic target, further research is warranted to explore novel interventions such as senolytics, anti-inflammatory agents, and metabolic modulators. The development of circulating biomarkers of vascular senescence (e.g., senescence-associated secretory phenotype [SASP] components and endothelial-derived extracellular vesicles) could enable early detection and risk stratification in individuals at high risk for VCI. Additionally, lifestyle modifications, including the Mediterranean diet, hold promise for delaying endothelial senescence and mitigating cognitive decline. In conclusion, cerebromicrovascular senescence is a key mechanistic link between atherosclerosis and cognitive impairment. Addressing microvascular aging as a modifiable risk factor through targeted interventions offers a promising strategy for reducing the burden of VCI and preserving cognitive function in aging populations.

## Introduction

Aging is a major risk factor for vascular cognitive impairment (VCI), a condition characterized by progressive cognitive decline due to cerebrovascular dysfunction [1–3]. Increasing evidence suggests that cerebromicrovascular senescence plays a crucial role in this process, contributing to cerebral blood flow (CBF) dysregulation, neurovascular coupling (NVC) impairment, blood–brain barrier (BBB) disruption, and genesis of cerebral microhemorrhages (CMHs) [4–11]. The accumulation of senescent endothelial cells in the cerebral microvasculature leads to persistent inflammation, oxidative stress, and impaired vascular integrity, which in turn exacerbates neurodegenerative processes [12, 13]. Recent findings by Lambert et al. [14] highlight the contribution of endothelial senescence in atherosclerosis-related cognitive impairment, providing a compelling framework for understanding the interplay between accelerated vascular aging and cognitive decline. Animal models of accelerated aging [6, 11, 15] and vascular pathology, such as atherosclerotic models, offer valuable opportunities to investigate the long-term impact of vascular dysfunction on cognitive outcomes. To accurately interpret the molecular and pathophysiological mechanisms underlying VCI, it is essential to recognize that vascular aging, much like the circulatory system itself, operates along a continuous anatomical spectrum, linking macrovascular pathology to microvascular dysfunction in the brain [16, 17].

## The continuum of vascular aging: linking macrovascular and microvascular pathologies

**Fig. 1:**
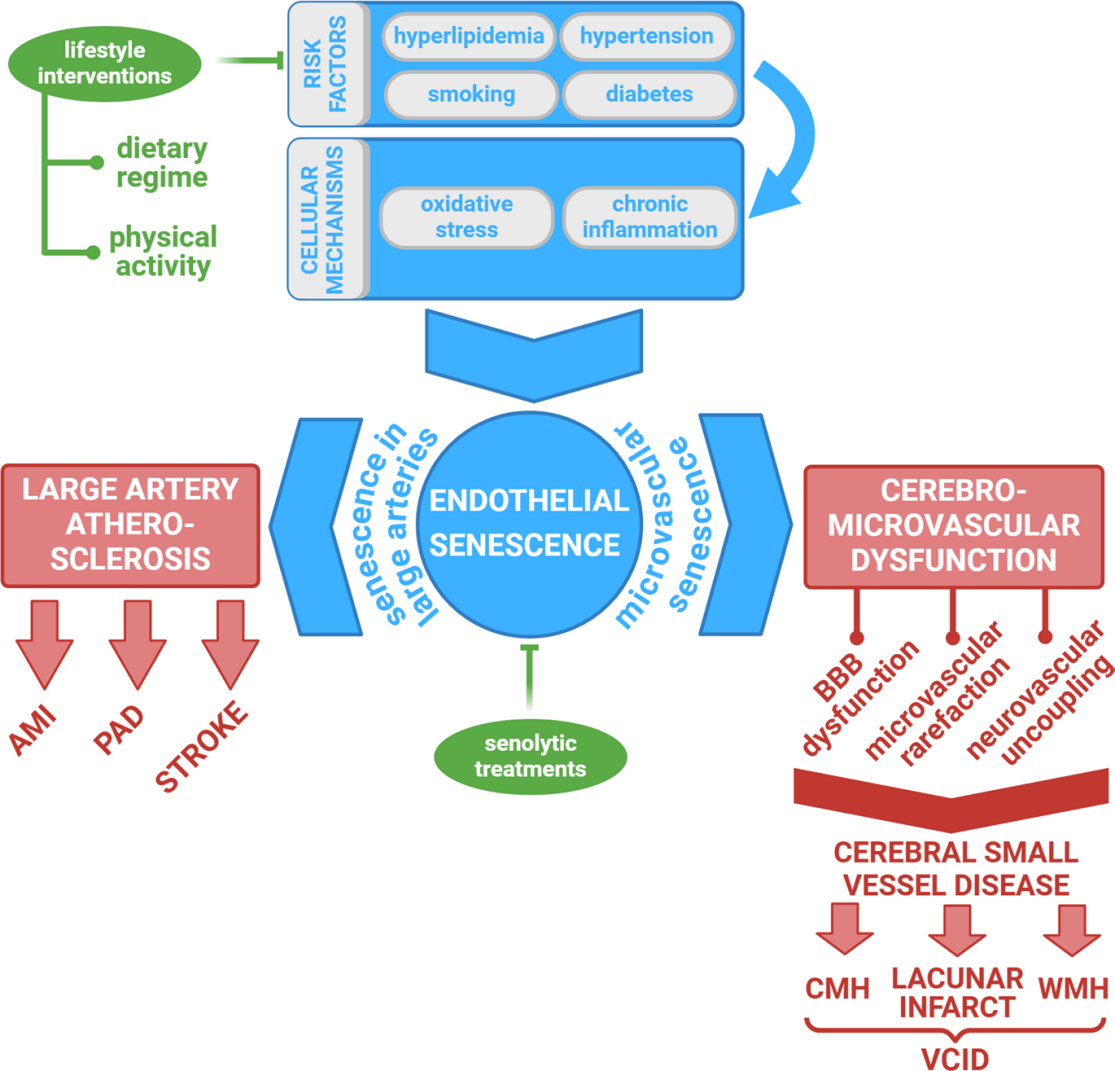
The continuum of vascular aging: endothelial senescence contributes to both atherogenesis in large vessels and microvascular pathologies leading to vascular cognitive impairment (VCID). The scheme illustrates the continuum of vascular aging, emphasizing the role of endothelial senescence in driving both macrovascular atherosclerosis and microvascular dysfunction. In large vessels, endothelial senescence promotes atherogenesis, while in the cerebral microvasculature, it leads to neurovascular uncoupling (NVC), cerebral blood flow dysregulation, blood–brain barrier (BBB) disruption, and subsequent neuroinflammation and microvascular rarefaction. Microvascular endothelial senescence contributes to cerebral small vessel disease (CSVD), manifesting as cerebral microhemorrhages (CMHs), lacunar infarcts, and white matter damage, all of which drive the pathogenesis of VCI. The interplay between macrovascular and microvascular aging underscores the critical importance of endothelial health in maintaining cognitive function. The figure also highlights potential therapeutic strategies, including senolytics and lifestyle modifications, aimed at mitigating endothelial senescence and preserving vascular and cognitive health across the vascular aging continuum. WMH: white matter hyperintensities

Vascular aging is not an isolated phenomenon affecting different vessel sizes separately but rather a continuum that spans from large arteries to the microvasculature [18, 19] (Fig. 1). This concept, supported by recent research, emphasizes that the pathological processes underlying atherosclerosis in large vessels also extend to the microcirculation, driving cerebrovascular dysfunction and cerebral small vessel disease (CSVD) [20–27] (Fig. 1). Shared risk factors and mechanisms—including the effects of pro-geronic circulating factors [28, 29], oxidative stress, chronic inflammation, endothelial dysfunction, and cellular senescence—contribute to both atherogenesis and microvascular aging, thereby accelerating the progression of VCI [18, 19, 30–33]. This common pathophysiological foundation renders the cerebral microcirculation particularly susceptible to dysfunction in patients with atherosclerosis, predisposing the brain to VCI [34, 35]. Recognizing this interplay underscores the importance of addressing vascular health across the entire vascular tree to mitigate the burden of cognitive decline in aging populations.

## Cerebromicrovascular endothelial senescence and cognitive decline

The cerebral microvasculature is essential for maintaining neuronal function by ensuring adequate blood supply, nutrient delivery, and waste clearance [12, 36–38]. However, with aging, endothelial cells progressively acquire a senescent phenotype, characterized by irreversible cell cycle arrest, structural alterations, and the secretion of a pro-inflammatory senescence-associated secretory phenotype (SASP) [18, 19, 39]. The SASP is characterized by the secretion of pro-inflammatory cytokines (e.g., IL-6, IL-1β, TNF-α), matrix metalloproteinases (MMPs), and reactive oxygen species (ROS), which create a pro-inflammatory microenvironment and contribute to pathological remodeling of the extracellular matrix, disruption of the endothelial barrier function, and promote endothelial dysfunction [18, 19, 40–42]. Additionally, senescent endothelial cells show telomere shortening, mitochondrial dysfunction, epigenetic alterations, and reduced proliferative capacity, all of which impair their ability to support vascular homeostasis [4, 32].

One of the critical consequences of endothelial senescence is the diminished production of nitric oxide (NO) [43, 44]. NO is a crucial mediator of vasodilation and NVC responses, and its deficiency leads to increased vascular stiffness, decreased cerebral blood flow, neurovascular uncoupling, and heightened susceptibility to ischemic injury [9, 45–47] (Fig. 1). Neurovascular uncoupling, a disruption of the normally synchronized relationship between neuronal activity and CBF, leads to regional hypoperfusion, particularly in high-energy-demanding brain regions such as the hippocampus and prefrontal cortex—key areas for memory and executive functions [46, 48, 49]. Over time, chronic hypoperfusion contributes to white matter damage, exacerbates neuroinflammation, and increases vulnerability to neurodegenerative pathologies [50–52]. Moreover, senescent endothelial cells exhibit structural abnormalities, such as increased permeability, disruption of tight junctions, and reduced expression of adhesion molecules necessary for maintaining vascular integrity [6, 19].

Senescent endothelial cells also play a direct role in BBB disruption (Fig. 1), a hallmark of VCI and Alzheimer’s disease [5, 6, 8, 53–55]. The loss of endothelial tight junction proteins (e.g., occludin, claudin-5, and ZO-1) results in increased vascular permeability, allowing toxic blood-derived molecules such as fibrinogen, albumin, and inflammatory mediators to enter the brain parenchyma [56–58]. This permeability breach activates resident microglia and astrocytes, triggering chronic neuroinflammation and synaptic dysfunction [56–58]. Additionally, senescent endothelial cells fail to support pericytes and astrocytes, which are essential components of the neurovascular unit, further compounding the BBB impairment [56, 59]. Emerging evidence also suggests that endothelial senescence contributes to microvascular rarefaction, the progressive loss of capillaries in the brain, which exacerbates cerebral hypoxia and oxidative stress [40, 60, 61]. This loss of microvascular density further compounds the metabolic deficits in aging brains and accelerates cognitive decline [61–63].

## The role of aging in promoting cerebromicrovascular endothelial senescence

Aging promotes endothelial senescence through a complex interplay of cellular and systemic mechanisms, ultimately impairing vascular function [64, 65] and contributing to neurovascular dysfunction [18, 19]. One of the primary drivers is oxidative stress, marked by excessive ROS production from both mitochondrial and non-mitochondrial sources, leading to widespread macromolecular damage, including oxidative DNA damage, lipid peroxidation, and protein modifications. ROS-mediated DNA damage triggers a senescence phenotype, wherein endothelial cells exhibit irreversible growth arrest, metabolic dysfunction, and a pro-inflammatory state known as the SASP [4, 18, 44, 66]. In addition to oxidative stress, systemic inflammation plays a crucial role in propagating endothelial senescence [18, 19]. Elevated levels of circulating pro-inflammatory cytokines, such as IL-6, TNF-α, and IL-1β, create a sustained inflammatory milieu that exacerbates vascular oxidative stress and accelerates endothelial dysfunction [43, 67]. This inflammatory feedback loop perpetuates endothelial cell activation, increases vascular permeability, and promotes the secretion of MMPs, which degrade the extracellular matrix and compromise vascular integrity [18, 19].

Epigenetic modifications further contribute to endothelial aging [18]. Age-associated changes in DNA methylation, histone acetylation, and chromatin remodeling disrupt gene expression patterns critical for endothelial homeostasis, leading to reduced expression of genes involved in vascular repair and increased expression of pro-senescent factors [18]. These epigenetic alterations impair endothelial resilience to stress and limit their capacity for repair and regeneration. Moreover, endothelial progenitor cell (EPC) dysfunction is a key hallmark of aging [68, 69]. EPCs, which normally replenish and repair damaged endothelial cells, exhibit reduced numbers and impaired regenerative capacity with aging [18, 70]. This decline limits the replacement of senescent endothelial cells and exacerbates vascular dysfunction [71].

Importantly, cardiovascular risk factors, including smoking, an unhealthy diet, physical inactivity, obesity, hypertension, diabetes mellitus, and hyperlipidemia, significantly exacerbate these aging-related processes [19, 72]. These factors further promote oxidative stress, systemic inflammation, endothelial injury, and mitochondrial dysfunction, accelerating endothelial senescence [19]. Chronic hyperglycemia, for example, induces non-enzymatic glycation of proteins (advanced glycation end-products, AGEs), which impair endothelial function and enhance ROS production [73]. Similarly, dyslipidemia and atherogenic lipoproteins contribute to vascular inflammation and endothelial dysfunction, compounding the deleterious effects of aging [74]. Collectively, these processes create a self-perpetuating cycle of accelerated endothelial aging, wherein oxidative stress, inflammation, and epigenetic alterations reinforce one another, ultimately leading to progressive microvascular dysfunction, BBB disruption, and neurovascular impairment. Addressing these mechanisms through targeted interventions, such as antioxidant therapies, anti-inflammatory strategies, lifestyle modifications, and senolytic treatments, holds promise for mitigating the effects of aging on cerebromicrovascular health and reducing the burden of VCI [46, 62, 75–80].

## Atherosclerosis as an accelerator of microvascular senescence

Atherosclerosis, traditionally considered a disease of large arteries, also affects the microvasculature, leading to an accelerated form of microvascular aging [18, 19, 31] (Fig. 1). Evidence from clinical and experimental studies has established a strong connection between atherosclerosis and VCI [21, 24]. Peripheral arterial disease (PAD) and carotid artery stenosis (CAS)—both manifestations of systemic atherosclerosis—are associated with an increased risk of CSVD, white matter hyperintensities, microhemorrhages, and ultimately, cognitive decline [26, 27, 81–86] (Fig. 1). These conditions contribute to BBB disruption, chronic cerebral hypoperfusion, and neuroinflammation, key mechanisms underlying VCI [21, 53, 87–90]. The relationship between peripheral atherosclerosis and cerebrovascular dysfunction underscores the concept of the continuum of vascular aging, linking systemic large artery disease to accelerated cerebromicrovascular aging and neurodegeneration.

The findings of Lambert et al. provide initial preclinical evidence supporting the concept of accelerated vascular aging in atherosclerosis. Their study demonstrated that atherosclerotic LDLr − / − ;hApoB100 + / + mice exhibit mild cognitive impairment, with significant learning deficits and partially compromised vascular function observed in both 6- and 12-month-old animals. Notably, senolytic treatment with ABT-263 improved macrovascular function in this model by enhancing endothelium-dependent vasodilation [14]. Importantly, ABT-263 also improved cognitive function and reduced the expression of key SASP factors—including ANGPTL2, PAI-1, and IL-6—in brain tissue, providing preliminary evidence that targeting senescent cells may mitigate the deleterious effects of atherosclerosis-induced vascular aging and associated cognitive decline [14].

Although this study highlights the contribution of atherosclerosis to cognitive decline and implicates the cerebrovasculature as a potential contributor, it does not specifically address the role of the cerebromicrovasculature in these pathological processes. Furthermore, the beneficial effects of ABT-263 were observed only in male LDLr − / − ;hApoB100 + / + mice, as evidenced by reduced astrogliosis and microgliosis and improved cognitive performance, suggesting that sex-dependent mechanisms may influence vascular senescence and cognitive outcomes in atherosclerosis [14, 91]. Understanding sex differences in endothelial senescence and cognitive decline is critical for developing targeted interventions. Several factors may underlie the differential response to ABT-263 between male and female mice. Hormonal influences, particularly the protective effects of estrogen, are known to enhance endothelial function, reduce oxidative stress, and suppress inflammation, potentially conferring greater vascular resilience in females [92]. Additionally, previous studies suggest that female mice may have a lower baseline burden of senescent cells or more efficient vascular repair mechanisms [92], which could modulate their response to senolytic therapy. Moreover, sex-dependent differences in immune system activation and inflammatory signaling may influence how vascular senescence contributes to cognitive impairment [92]. Given that microglial activation, astrocytic reactivity, and peripheral immune responses differ between sexes, these factors may also shape the variability in treatment efficacy [92]. Further research is needed to elucidate the molecular basis of these sex differences, determine how senescence pathways are differentially regulated, and identify sex-specific therapeutic approaches to mitigate vascular aging and cognitive decline. Additionally, De Montgolfier et al. demonstrated that in the same LDLr − / − ;hApoB100 + / + model, the presence of systolic hypertension induces neurovascular unit disruption by reducing endothelial nitric oxide synthase (eNOS) expression and decreasing collagen IV content in the cerebrovascular basement membrane, further compromising vascular integrity and potentially exacerbating cognitive decline in atherosclerosis model mice [93].

Furthermore, future studies should also elucidate the role of senescence in vascular smooth muscle cells (VSMCs) [94–99] and pericytes [58, 59, 100–106] in neurovascular aging and VCI. Age-related dysfunction of VSMCs contributes to impaired vasodilation, increased vascular stiffness, and loss of cerebrovascular autoregulation [9, 107], exacerbating cerebral hypoperfusion and increasing susceptibility to both rupture (genesis of cerebral microhemorrhages [4, 94, 108–110]) and ischemic injury. Similarly, pericyte degeneration plays a pivotal role in blood–brain barrier (BBB) disruption, as pericytes are essential for endothelial barrier integrity, capillary stabilization, and neurovascular communication [58, 59, 100–106]. The age-associated decline in pericyte function has been linked to increased BBB permeability, heightened neuroinflammation, and progressive white matter damage, further contributing to cognitive impairment [58, 59, 100–106]. By addressing senescence in these critical vascular cell types, future research can provide a more comprehensive understanding of microvascular aging and its impact on neurovascular function, ultimately guiding the development of targeted interventions to preserve cerebrovascular health and prevent VCI.

Collectively, these findings underscore the critical role of vascular senescence in atherosclerosis-associated cognitive impairment, highlighting vascular aging as a key therapeutic target. However, more detailed and mechanistic studies are essential to fully unravel the pathophysiological contributions of the cerebromicrovasculature to cognitive decline in atherosclerosis and to develop effective interventions.

To establish mechanistic evidence supporting the role of atherosclerosis-induced microvascular endothelial senescence as a key contributor to VCI, future studies should utilize preclinical models of atherosclerosis to investigate critical aspects, including cerebromicrovascular endothelial function, NVC responses, BBB integrity, capillary density, neuroinflammatory markers, and CMH burden. Given the time-dependent progression of both atherosclerotic pathology and the potential therapeutic window for senolytic interventions, it is essential that future research incorporates longitudinal in vivo tracking experiments to monitor disease progression and treatment responses over time. In addition to elucidate these mechanisms, future studies should also explore novel senolytic therapeutic strategies targeting microvascular senescence and assess their impact on these endpoints. To effectively evaluate the burden of endothelial senescence and the efficacy of these treatments, advanced methodologies such as genetic models for senescent cell identification, scRNA-seq, and spatial transcriptomics should be employed. These approaches will allow for precise characterization of cellular heterogeneity and the molecular pathways underlying microvascular aging, enabling the development of targeted interventions to combat atherosclerosis-induced cerebrovascular dysfunction.

To effectively translate these findings into clinical practice, efforts should focus on developing biomarkers of vascular senescence that can serve as early indicators of cerebrovascular aging. Circulating endothelial senescence markers, such as SASP components and endothelial-derived extracellular vesicles, could be utilized for early detection and risk stratification of individuals with atherosclerotic vascular diseases at high risk for VCI.

Ongoing clinical studies in different contexts investigate circulating biomarkers of cellular senescence, such as SASP components (e.g., IL-6, PAI-1, ANGPTL2) and endothelial-derived extracellular vesicles, as potential indicators of cerebrovascular aging [111–115]. However, several challenges remain, including the validation of these biomarkers in large, well-characterized patient cohorts, the specificity of these markers for vascular senescence versus generalized inflammation, and the feasibility of implementing high-throughput screening in clinical settings. Addressing these barriers is essential for integrating vascular senescence biomarkers into routine risk stratification and early intervention strategies for VCI. Moreover, postmortem analyses of brains from individuals with systemic atherosclerosis and VCI should be conducted to assess endothelial senescence markers and correlate these indices with microvascular rarefaction and biomarkers of BBB disruption. Such investigations would provide direct histopathological evidence linking macrovascular disease to microvascular aging, further reinforcing the concept of vascular aging as a systemic process. These studies will be crucial for bridging the gap between preclinical findings and human disease pathology, ultimately advancing the development of diagnostic and therapeutic strategies for vascular cognitive impairment.

Additionally, lifestyle and pharmacological interventions targeting endothelial senescence should be explored for their potential to delay or mitigate VCI progression. Strategies such as dietary interventions (e.g., the Mediterranean diet), structured exercise programs, and pharmacological agents with senolytic or senomorphic properties offer promising avenues for intervention [6, 10, 19, 44, 76, 116]. Further clinical trials are needed to assess the efficacy of these strategies in preserving cerebrovascular function and reducing cognitive decline in aging populations.

## Conclusion

Cerebromicrovascular senescence is a fundamental driver of cerebrovascular dysfunction and cognitive decline in aging, contributing to neurovascular uncoupling, blood–brain barrier disruption, and cerebral microhemorrhages. The concept of the continuum of vascular aging, linking macrovascular atherosclerosis to microvascular senescence, provides a crucial framework for understanding vascular contributions to VCI and neurodegeneration. Preclinical findings, including those from Lambert et al. [14], underscore the role of atherosclerosis-induced microvascular senescence in exacerbating cognitive impairment [16, 17]. Their study provides additional evidence that senolytic therapy may hold promise for reversing endothelial senescence and microvascular dysfunction [4, 10, 64, 117, 118] and improving cognitive function, warranting further investigation in translational models. The implementation of cutting-edge technologies such as scRNA-seq and spatial transcriptomics will allow for a deeper understanding of microvascular aging at the molecular level, facilitating the development of translationally relevant targeted therapies.

To advance clinical applications, the development of vascular senescence biomarkers is essential for early detection and intervention in individuals at risk for VCI. Future research should also focus on lifestyle and pharmacological interventions aimed at mitigating endothelial aging, including dietary strategies (e.g., the Mediterranean diet, time-restricted eating), exercise, and senolytic/senomorphic therapies. Addressing cerebromicrovascular senescence as a modifiable risk factor holds significant potential for reducing the burden of vascular cognitive impairment and preserving brain health in aging populations.
